# Electrical Stimulation in the Human Cochlea: A Computational Study Based on High-Resolution Micro-CT Scans

**DOI:** 10.3389/fnins.2019.01312

**Published:** 2019-12-05

**Authors:** Siwei Bai, Jörg Encke, Miguel Obando-Leitón, Robin Weiß, Friederike Schäfer, Jakob Eberharter, Frank Böhnke, Werner Hemmert

**Affiliations:** ^1^Department of Electrical and Computer Engineering, Technical University of Munich, Munich, Germany; ^2^Munich School of Bioengineering, Technical University of Munich, Garching, Germany; ^3^Graduate School of Biomedical Engineering, University of New South Wales, Sydney, NSW, Australia; ^4^Medizinische Physik and Cluster of Excellence Hearing4all, Universität Oldenburg, Oldenburg, Germany; ^5^Graduate School of Systemic Neurosciences, Ludwig Maximilian University of Munich, Planegg, Germany; ^6^Department of Otorhinolaryngology, Klinikum rechts der Isar, Munich, Germany

**Keywords:** cochlear implant, computational model, finite element analysis, electrical stimulation, auditory nerve fibers, model reconstruction

## Abstract

**Background:** Many detailed features of the cochlear anatomy have not been included in existing 3D cochlear models, including the microstructures inside the modiolar bone, which in turn determines the path of auditory nerve fibers (ANFs).

**Method:** We captured the intricate modiolar microstructures in a 3D human cochlea model reconstructed from μCT scans. A new algorithm was developed to reconstruct ANFs running through the microstructures within the model. Using the finite element method, we calculated the electrical potential as well as its first and second spatial derivatives along each ANF elicited by the cochlear implant electrodes. Simulation results of electrical potential was validated against intracochlear potential measurements. Comparison was then made with a simplified model without the microstructures within the cochlea.

**Results:** When the stimulus was delivered from an electrode located deeper in the apex, the extent of the auditory nerve influenced by a higher electric potential grew larger; at the same time, the maximal potential value at the auditory nerve also became larger. The electric potential decayed at a faster rate toward the base of the cochlea than toward the apex. Compared to the cochlear model incorporating the modiolar microstructures, the simplified version resulted in relatively small differences in electric potential. However, in terms of the first and second derivatives of electric potential along the fibers, which are relevant for the initiation of action potentials, the two models exhibited large differences: maxima in both derivatives with the detailed model were larger by a factor of 1.5 (first derivative) and 2 (second derivative) in the exemplary fibers. More importantly, these maxima occurred at different locations, and opposite signs were found for the values of second derivatives between the two models at parts along the fibers. Hence, while one model predicts depolarization and spike initiation at a given location, the other may instead predict a hyperpolarization.

**Conclusions:** Although a cochlear model with fewer details seems sufficient for analysing the current spread in the cochlear ducts, a detailed-segmented cochlear model is required for the reconstruction of ANF trajectories through the modiolus, as well as the prediction of firing thresholds and spike initiation sites.

## 1. Introduction

The cochlea in the inner ear is a complex three-dimensional structure, where sound is coded by the sensory hair cells into electrical impulses traveling along the auditory nerve to the brain. These hair cells are easily damaged, which leads to permanent hearing loss. Cochlear implants (CIs) are surgically-implantable biomedical devices that bypass the sensory hair cells and directly excite the remaining fibers of the auditory nerve with electric current. They are capable of restoring a surprisingly large degree of auditory perception to patients that are severe-to-profoundly deaf. Up to the year of 2012, there were more than 325,000 CI recipients all over the world, and more than 100,000 CI users in Europe (De Raeve and van Hardeveld, 2013), which were about 200 implanted patients per million inhabitants. However, this only accounts for 7% of all adults with hearing impairment that could benefit from a CI in Europe (De Raeve and van Hardeveld, 2013). In addition, the estimated prevalence of permanent bilateral hearing impairment among newborns varies from 0.1 to 0.4 % (Fortnum et al., 2001), among which 45% are considered potential CI candidates (De Raeve and van Hardeveld, 2013).

As the human cochlea is deeply embedded inside the temporal bone, direct measurements of electrical potential or current along the auditory nerve fibers are not readily feasible. Computational cochlear models have been extensively utilized to simulate current spread in the cochlea and neuronal excitation, and provided useful insights. For instance, it has been demonstrated that the anatomical structure, such as the tapering spiral feature of the cochlea (Briaire and Frijns, 2000), the conductivity of the bone and other structures (Kalkman et al., 2014; Wong et al., 2015; Malherbe et al., 2016) and the inclusion of a head model (Malherbe et al., 2016), influence the current spread as well as the neural excitation pattern. In addition, the location of electrode array relative to the cochlear wall has also a strong effect on the distribution of electrical current as well as the excitation pattern of the auditory nerve (Frijns et al., 2001; Hanekom, 2001; Malherbe et al., 2016).

Nevertheless, due to limitations in image acquisition and model reconstruction, many detailed features of the cochlear anatomy have not been included in existing models. These features include the microstructures inside the modiolar bone, where spiral ganglion neurons (SGNs) reside and neural fibers as well as blood vessels run through. As a result, the peripheral processes of the auditory nerve have been conventionally modeled as a smooth sheet extending into the main trunk of the nerve without taking into account the bone porosity (Finley et al., 1990; Frijns et al., 2001; Hanekom, 2001, 2005; Rattay et al., 2001a; Choi et al., 2005; Kalkman et al., 2014, 2015; Malherbe et al., 2016; Mangado et al., 2016; Nogueira et al., 2016). Moreover, during the auditory nerve fiber reconstruction in most studies, both the dendritic ends and the ganglion cell bodies were considered evenly distributed around the central axis of the modiolus, the auditory nerve fibers (ANFs) were then reconstructed by applying spline interpolation between the dendritic end and the ganglion cell body, and a spline extrapolation beyond the ganglion cell (Frijns et al., 2001; Hanekom, 2001, 2005; Kalkman et al., 2014, 2015; Malherbe et al., 2016; Mangado et al., 2016; Nogueira et al., 2016). It has been suggested in Kalkman et al. (2015) that a model with grouped ganglion cell bodies, similar to reality, results in a more focussed excitation pattern than a model with evenly distributed cell bodies. Hence, it is necessary to investigate the influence of the modiolar bone porosity on the electrical current spread as well as the excitation pattern of the auditory nerve.

The excitation pattern of ANFs is in general predicted by the implementation of multi-compartment cable models (Rattay et al., 2001b, 2013; Briaire and Frijns, 2005; Smit et al., 2010; Potrusil et al., 2012). The cable models incorporate neural compartmental impedances that affect the amplitude of intracellular potential generated within neural compartments in response to external stimulus delivered by CI electrodes. Nevertheless, there exist several ANF models in the literature with varied morphological or ionic channel properties. Choosing the appropriate cable model for a given computational study is difficult, as different models does not necessarily respond the same way to a given stimulus (Bachmaier et al., 2019). Consequently, most models adopted in existing studies were chosen without a specific reason or by inheritance. Rattay (1986) has shown that for a stimulation of an axon with an extracellular electrode, the activating function $f=\frac{d}{4\rho_{i}c}\cdot \frac{\partial^{2}V}{\partial x^{2}}$ (*d*, ρ_*i*_, and *c* represent, respectively the fiber diameter, the axomplasmatic resistivity and capacity per unit length) predicts the initiation of an action potential. With the assumption of *d*, ρ_*i*_, and *c* remaining constant, activation is then correlated to the second spatial derivative of external voltage $\frac{\partial^{2}V}{\partial x^{2}}$. We thus in this study decided to adopt the activating function for the analysis of spike initiation sites, before we implement more complex multi-compartment models.

In this paper, we introduced a new three-dimensional (3D) model of the implanted human cochlea from a set of high-resolution μCT scans using the finite element (FE) method; this model managed to capture the intricate microstructures inside the modiolar bone. Subsequently, we validated simulation results against intracochlear measurements, and compared the detailed model to a simplified model without these microstructures. Due to the structural irregularity inside the modiolus of the detailed model, conventional methods to generate ANFs are not applicable. We hereby also developed a new algorithm to reconstruct ANFs within the 3D cochlear model.

## 2. Methods

### 2.1. FE Model Reconstruction

The μCT scans of a human cadaveric temporal bone with an inserted dummy electrode (pure silicone, without platinum alloy wires or contacts) were acquired by the Department of Otorhinolaryngology at the Rechts der Isar Hospital, with an isotropic voxel size of 5.9 μm and a spatial resolution of 3, 000 × 3, 000 × 2, 752 voxels (Braun et al., 2012). The scans were initially processed to enhance the contrast and the edges between different tissues. Due to limitation of the computational memory, the field of view of the scans were subsequently rescaled to include only the cochlea and its immediate surroundings, and later downsampled to an isotropic resolution of 9.6 μm with a spatial resolution of 930 × 930 × 1, 014 voxels.

The segmentation of the μCT scans was performed in 3D Slicer (Version 4.6) (Kikinis et al., 2014), an open-source platform for medical image processing. In 3D Slicer, each tissue compartment was assigned a label map. To generate a label map, a threshold was chosen for the gray level of the pixel intensity at a single slice to automatically select most of the desired tissue, and a paintbrush was used to manually modify the selection. This procedure was repeated at every second or third slice until the end of the dataset, and an interpolation method was later used to create a full segmentation by automatically connecting the sparse set of contours. A paintbrush was then chosen again to modify the tissue map until a desired accuracy was met. The segmented tissue compartments from the μCT scans are bony labyrinth, cochlear canal and cochlear nerve. A surface triangular mesh was generated for every compartment.

The T1-MRI scans of a human head were acquired with an isotropic voxel size of 1 mm. After the enhancement of the image contrast, the head scans were automatically segmented into three compartments, i.e., scalp, skull, and brain, in BrainSuite (Shattuck and Leahy, 2002), an open-source software specialized in processing MRI head scans. The surface meshes of the cochlear model and head model were then imported into Blender, an open-source platform for 3D computer graphics. The coordinate systems of both models were aligned in Blender, so that the cochlear model was embedded in the head model at the petrous part of the left temporal bone. Further processing was subsequently performed on all surface meshes in Geomagic Wrap (3D Systems, SC, USA) to increase the mesh quality and smoothness. The procedures included removing non-manifold edges, splitting self-intersecting triangles, reducing edge crease, smoothing spikes, and repairing holes.

**Figure 1:**
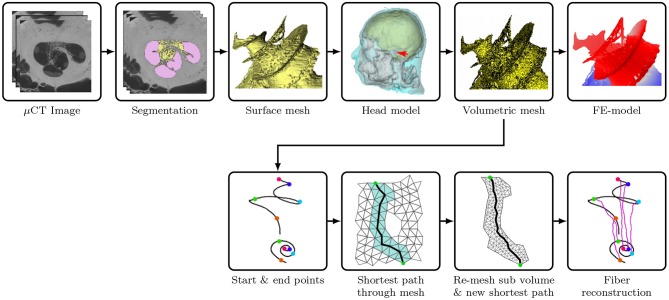
Illustration of the procedures to reconstruct the FE cochlear model from μCT scans of a piece of human cadaveric temporal bone. (Flowchart reconstruction both FE and nerve—JE). Illustration of the procedures to reconstruct the auditory nerve fibers in the auditory nerve: (1) find the shortest path through the FE Mesh; (2) select sub-volume surrounding the mesh, and remesh the sub-volume to find a new shortest path; (3) generate a multi-compartment model based on the fiber trajectory.

**Figure 2:**
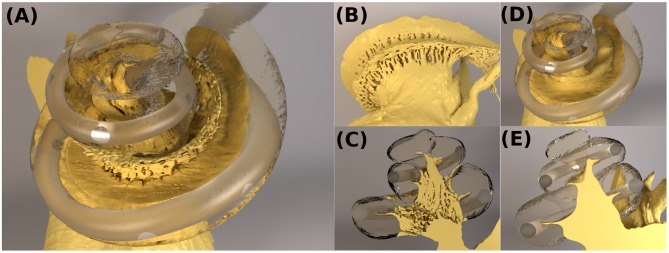
**(A–C)** The cochlear model “ORI” with a detailed-segmented auditory nerve geometry; **(D,E)** The cochlear model “SIM” with a simplified nerve model, whose fine details through the Rosenthal's canals were removed.

Afterwards, all surface meshes were transferred to ANSYS ICEM CFD (ANSYS, PA, USA). After defining edges at the intersections between compartments and at the desired electrode contact locations, the tetrahedral volumetric mesh was generated with appropriate meshing and coarsening parameters. The aforementioned model reconstruction procedures are demonstrated in Figure 1. The volumetric mesh, with 21,937,778 elements, was exported to COMSOL Multiphysics (COMSOL AB, Sweden), a cross-platform FE solver, for the simulation of electrical stimulation. The geometry of the cochlear model (cochlear canal, auditory nerve and CI electrode) is presented in Figures 2A–C.

In the segmented cochlear model (named “ORI”), the fine details of microstructures through the Rosenthal's canals were captured, as illustrated in Figure 2C. In order to investigate the influence of these microstructures, the auditory nerve model was subsequently modified by removing all of the fine details inside the modiolar bone, and the resulting simplified cochlear model was named “SIM.” The geometry of SIM (cochlear canal, simplified auditory nerve and CI electrode) is displayed in Figures 2D,E. This model resulted in a volumetric mesh of 26,848,015 elements.

The electric potential *V* in the model was calculated using Laplace's equation: ∇ · (−σ∇*V*) = 0, where σ is the electric conductivity, and ∇ is the nabla partial differentiation operator given by $\nabla \equiv \left(\frac{\partial }{\partial x},\frac{\partial }{\partial y},\frac{\partial }{\partial z}\right)$. The electrical conductivity of model compartments (Bai et al., 2012; Malherbe et al., 2016) is shown in Table 1. The electric permittivity of biological tissues in the model is neglected under quasi-static approximation (Malmivuo and Plonsey, 1995).

**Table 1:** The electrical conductivities of all compartments in the cochlear model.

**Structure**	**Conductivity / S m-1**
Scalp	0.33
Skull	0.013
Brain	0.2
Bony labyrinth	0.013
Silicone electrode	0
Cochlear canal	1.43
Auditory nerve	0.3333

*All conductivity values were adapted from Malherbe et al. (2016), except for the bone (Bai et al., 2012)*.

### 2.2. CI Electrode Design and Stimulation Scheme

The dummy CI electrode in the cadaveric temporal bone was also reconstructed from the μCT scans. The electrode was inserted through the round window into the scala tympani. The electrode then punctured the basilar membrane at approximately 270° and traveled along the scala vestibuli, until it stopped at an approximately 720° angle into the cochlear canal, as shown in Figure 2A. This translocation likely resulted from changes in the mechanical properties of tissues in the cadaveric bone, which became more rigid. Nevertheless, translocation may also occur in clinical settings (Holden et al., 2013; Risi, 2018).

The conductivity of the silicone CI electrode was assigned to be zero. The electrode contacts were arranged based on the MED-EL (Innsbruck, Austria) Standard twelve-contact-pair design, with a contact radius of approximately 0.18 mm and a centre-centre distance of approximately 2.4 mm. The current-controlled stimulation scheme was monopolar with a total electric current of 1 mA from an electrode contact pair; all other pairs were inactive at floating potentials, with the net current being zero. The stimulating electrodes were numbered from the base to the apex of the cochlea. The CI reference electrode with a radius of approximately 1 cm was set as ground and placed extracochlearly on the left temporal bone of the skull, superior, and posterior to the left external acoustic meatus.

### 2.3. Nerve Fiber Reconstruction

In Blender, a spiral was defined along the entire outer edge of osseous spiral lamina (25.003 mm). This curve was, representing the synaptic ending of the peripheral axon, used to derive the starting points for all fibers. A second spiral was created by projecting the starting curve onto the plane, where the base of the truncated auditory nerve sits. The projected spiral was shrinked to fit in the base, and subsequently rotated by 45°. This curve then acted as the basis of the end points of all fibers. On both of the spirals, the spacial coordinates of 400 evenly-spaced seed points, including the endpoints of the spirals, were exported.

ANFs were reconstructed based on these seed points with a program written in Python. The seed points were firstly mapped onto the closest nodes of the FE mesh of the auditory nerve. The shortest path through the FE mesh between each pair of points on the start and end curves was calculated by using Dijkstra's algorithm, as shown in Figure 1. Later, a sub-volume was extracted around each of the approximated fiber trajectories, and was subsequently remeshed with a finer resolution in order to smooth the fiber. The final fiber trajectory was gained by re-applying Dijkstra's algorithm on every remeshed sub-volume.

**Figure 3:**
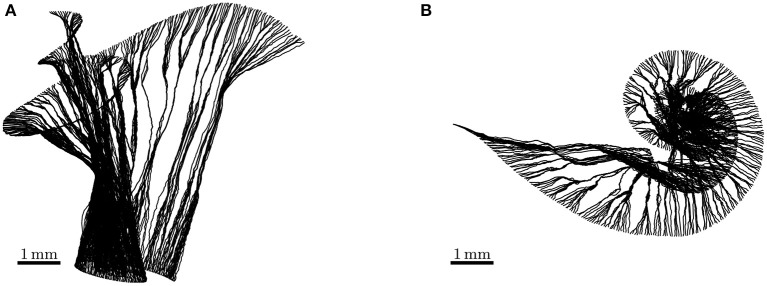
Side view **(A)** and top view **(B)** of auditory nerve fibers reconstructed from the detailed-segmented auditory nerve in the cochlear model “ORI.” Their lengths range from 5.520 to 8.151 mm.

We reconstructed fibers using the FE meshes of the auditory nerve in the ORI model. The reconstructed ANFs are illustrated in Figure 3, and the fiber lengths lay within the ranges of 5.520–8.151 mm. As the SGN peripheral axon has an average length of 1.5 mm (Spoendlin and Schrott, 1989; Rattay et al., 2001b), and the soma diameter is recently reported to be 20 μm (Potrusil et al., 2012), these reconstructed ANF trajectories represented the peripheral axon, soma, and part of central axon of the SGNs.

For data analysis, the electrical potential data for both ORI and SIM were extracted using the coordinates of ANFs acquired from the detailed-segmented auditory nerve in the ORI model. We then calculated the first and second derivatives of electric potential along the fiber direction. As the derivatives of the raw voltage data exhibited large peak values that would have been smoothed by the nerve fibers, we applied a low-pass filter derived from the length constant of myelinated axons of spiral ganglion cells to the voltage data, before calculating the derivatives. A similar approach can be found in Zierhofer (2001), where the author approximated the steady-state solution to the cable equation with a convolution product of the second spatial derivative of the external potential and a spatial low-pass filter depending on the length constant of the fiber. The length constant λ is defined as

(1) $$\begin{array}{l} \lambda =\sqrt{\frac{\rho_{m}\cdot a}{2\rho_{i}}}, \end{array}$$

where the transmembrane resistivity ρ_*m*_ is 1 kΩ · cm^2^ per myelin layer for 80 layers, the intracellular resistivity is ρ_*i*_ 0.05 kΩ · cm, and the axonal radius *a* is 1 μm (values taken from Rattay et al., 2001b). The first derivative $\frac{\partial V}{\partial x}$ was approximated by

(2) $$\begin{array}{l} V_{k}^{\prime }\approx \frac{V_{k+1}-V_{k}}{\left|r_{k,k+1}\right|}, \end{array}$$

where *k* represents the *k*th node on an individual fiber, $V_{k}^{\prime }$ is the first derivative of *V* at the *k*th node, and |*r*_*k, k*+1_| is the distance between the *k*th and *k*+1th nodes on the fiber. Using the finite difference method, the second derivative $\frac{\partial^{2}V}{\partial x^{2}}$ at the *k*th node can be approximated as.

(3) $$\begin{array}{l} V_{k}^{\prime \prime }\approx \frac{\frac{V_{k+1}-V_{k}}{\left|r_{k,k+1}\right|}-\frac{V_{k}-V_{k-1}}{\left|r_{k-1,k}\right|}}{\frac{\left|r_{k-1,k+1}\right|}{2}} \end{array}$$

### 2.4. Intracochlear Potential Measurements

Intracochlear potentials were measured in 10 CI users (16 ears) using the telemetry system of the CIs (Zierhofer, 2000). Table 2 lists relevant information on all CI subjects in this experiment. In the present experiment, biphasic pulses of 40 μs, with an inter-phase gap of 2.1 μs and with the cathodic (negative) phase leading, were used as stimuli. The voltage at the measuring electrode was recorded by the telemetry system at the end of the anodic phase in the stimulating electrode. The pulses had an amplitude of 50 CU (1 CU ≈ 1 µA).

**Table 2:** Information on CI subjects participating the intracochlear measurement.

**Subject**	**Age**	**Ear**	**Hearing difficulty**	**CI type**	**Implant use**
ID2	55 y/o	L	From birth	Pulsar	12 years
		R	From birth	Sonata	10 years
ID3	64 y/o	L	20 years	Sonata	5 years
		R	20 years	Sonata	4 years
ID4	58 y/o	L	56 years	Pulsar	11 years
		R	56 years	Pulsar	10 years
ID5	68 y/o	L	27 years	Pulsar	12 years
		R	27 years	Pulsar	6 years
ID6	64 y/o	L	32 years	Concerto	2 years
		R	32 years	Synchrony	8 years
ID7	56 y/o	L	44 years	Synchrony	3 years
ID8	42 y/o	L	From birth	Concerto	4 years
ID10	77 y/o	L	30 years	Synchrony	20 years
		R	32 years	Synchrony	10 years
ID11	52 y/o	L	17 years	Synchrony	10 months

*All CIs are products from MED-EL (Innsbruck, Austria)*.

A Research Interface Box (RIB2, University of Innsbruck) was used to communicate with the implants. Customized software written in Python was used to generate the stimuli and record the telemetry results. The MED-EL impedance field telemetry (IFT) system used a track-and-hold circuit, which followed the voltage only during anodic phases and held the voltage at their end. This measured voltage was then output as 2,048 bits of adaptive sigma-delta-modulated data (Zierhofer, 2000). Subsequently, the voltage value was obtained by averaging and multiplying by a factor provided by the manufacturer. A more detailed description and characterization of the IFT system can be found in Neustetter (2014) for a more detailed description and characterization of the IFT system.

A full voltage spread matrix was measured, meaning that all (active) electrodes were measured against all other electrodes, respectively. Some data points were missing due to the electrodes being deactivated or showing clearly outlying (very high) impedances, which indicated bad contacts. This was mostly the case for the most basal electrodes, which indicated these electrodes were not completely inside the cochlea.

Measurements presented in this work were conducted prior to other experiments in our workgroup. All subjects gave their informed consent and received monetary compensation for their participation. Measurements were conducted in accordance to the Declaration of Helsinki, and were approved by the medical ethics committee of the Klinikum rechts der Isar (Munich, 2126/08).

## 3. Results

### 3.1. Model Validation

**Figure 4:**
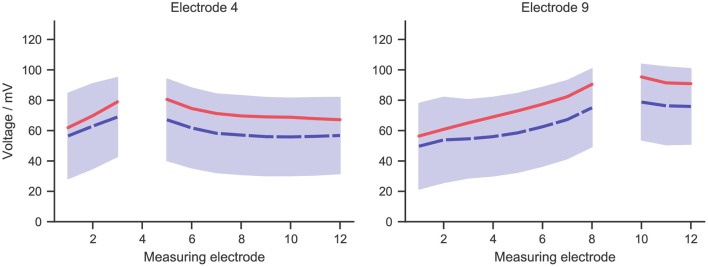
The mean (dashed blue line) and standard deviation (blue zone) of intracochlear potential measurement data for two exemplary electrodes: 4 and 9. The solid red line represents data from the simulation, whose stimulating current was adjusted to the same value as in the measurement, i.e., 50 μA.

For measurements at any implant electrode, a broad range of values was observed within cochlear implant subjects. This was shown in Figure 4, which presents the mean (in dashed blue line) and standard deviation of measurements at two exemplary electrodes. Measurements at the stimulating electrodes were left out, as the model did not account for the electrode-lymph interface. Simulation data with the stimulating current adjusted to the same value as in the experiment was also shown in the figure in solid red line. The shape of the simulated curve matched the measurements closely, and the simulated values fell within the range of measurement data, although the simulation slightly overshot the mean curve.

### 3.2. Stimulation Profile of the Detailed Model

**Figure 5:**
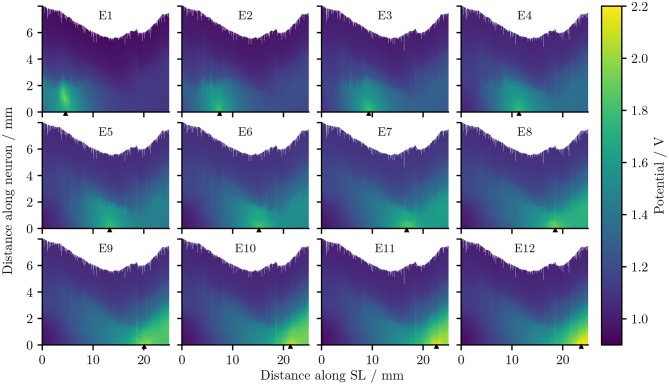
The electrical potentials along the reconstructed fibers within the detailed-segmented cochlear model. The stimulating electrode for each plot is specified at the top, and its location along the edge of spiral lamina is marked by the black solid triangles on the *x*-axis. “SL” stands for the outer edge of osseous spiral lamina.

Figure 5 describes the electrical potential profile, extracted from ORI, i.e., the detailed-segmented cochlear model, along the 400 reconstructed fibers arranged from the base to the apex of the cochlea. As is observed in the plots, the maximal potential value of each simulation appeared in proximity to the stimulating electrode, whose position is indicated by a small triangle in Figure 5. The maximal value was also located in most situations close to, if not at, the synaptic ending of peripheral axons (i.e., tip of the ANFs); the exception occurred when E1, i.e., the most basal electrode, was the stimulating electrode, and the maximal potential value showed up at approximately 1 mm from the nerve fiber tip. The reason is that in the 3D model E1 was close to the medial wall of scala tympani, whereas all other electrodes were near the lateral wall of cochlea.

**Figure 6:**
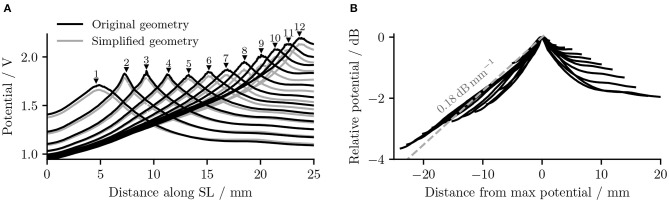
**(A)** The electrical potentials (in absolute value) along the edge of the spiral lamina within the “original” detailed-segmented and “simplified” cochlear models. The number and the black solid triangle above each potential line, respectively, specify the stimulating electrode used and its location along the spiral lamina to produce this specific line. “SL” stands for the outer edge of osseous spiral lamina. **(B)** The normalized electrical potentials along the edge of the spiral lamina within the original detailed-segmented model. Each potential line was normalized to its maximal value.

**Figure 7:**
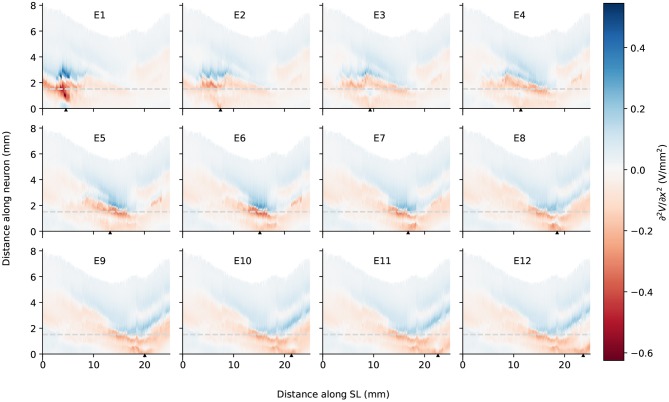
The second spatial derivative of electrical potential along the fiber direction within the detailed-segmented cochlear model. The stimulating electrode for each plot is specified at the top, and its location along the edge of spiral lamina is marked by the black solid triangles on the *x*-axis. The gray line in each plot indicates the soma location. “SL” stands for the outer edge of osseous spiral lamina.

It is also obvious from Figure 5 that as the stimulating electrode shifted toward the apex, the extent covered by a higher potential grew larger, which suggests the stimulation became less discriminative; meanwhile, the maximal potential value became larger as the stimulating electrode was shifted upwards, despite that the electrical stimulation current remained the same for all electrodes. This is also depicted in Figure 6A, which compares the electrical potential in absolute value along the edge of the spiral lamina, i.e., the synaptic ending of peripheral axons for all stimulating electrodes. When these electrical potentials at the synaptic endings were normalized to their respective maximum, as in Figure 6B, it showed a converged decline toward the base at a speed of approximately 0.18 dB/mm; in comparison, the decay toward the apex was slower and flattened out at a different level for each electrode. The current conservation was also reflected in Figure 7, which illustrates the second spatial derivative of electrical potential along the fiber direction, but the effect was not as prominent as in the electrical potential profile.

### 3.3. Comparison Between the Two Models

**Figure 8:**
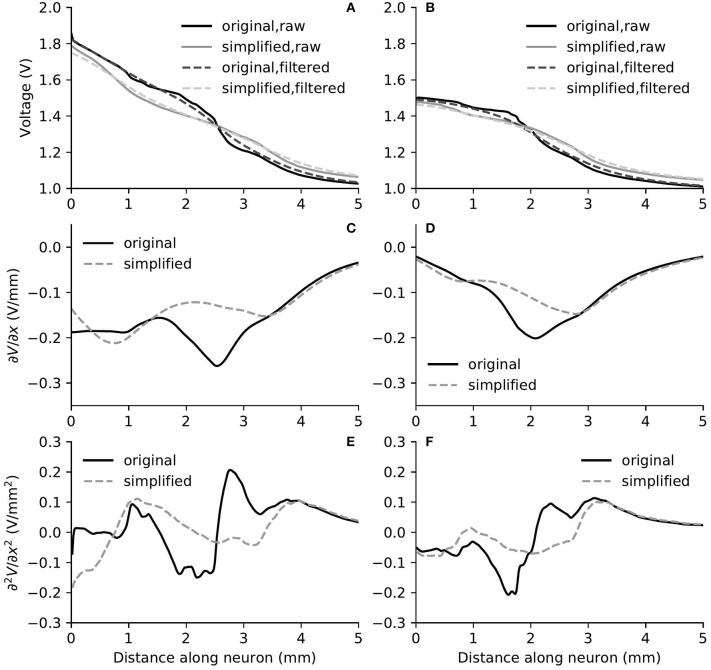
The electric potential **(A,B)**, as well as the first **(C,D)** and second **(E,F)** derivatives of filtered electric potential along the fiber direction for two example fibers (truncated at 5 mm) within the “original” detailed-segmented and “simplified” cochlear models, when the stimulating electrode was E3. The electric potential **(A)**, first **(C)** and second **(E)** derivatives on the left column were taken from the fiber 9.31 mm on the spiral lamina away from the base, which was also the closest fiber to the stimulating electrode. The electric potential **(B)**, first **(D)** and second **(F)** derivatives on the right column were taken from the fiber 12.44 mm away from the base.

**Figure 9:**
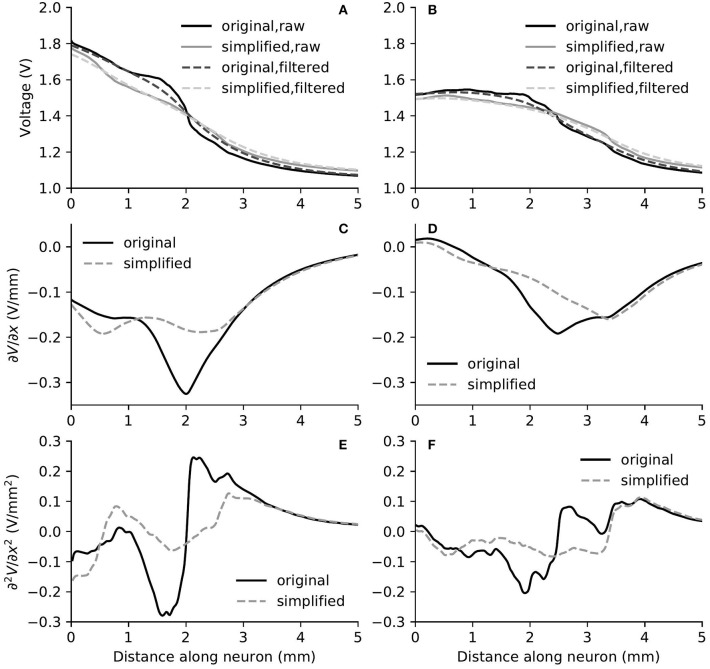
The electric potential **(A,B)**, as well as the first **(C,D)** and second **(E,F)** derivatives of filtered electric potential along the fiber direction for two example fibers (truncated at 5 mm) within the “original” detailed-segmented and “simplified” cochlear models, when the stimulating electrode was E5. The electric potential **(A)**, first **(C)** and second **(E)** derivatives on the left column were calculated on the fiber 13.27 mm on the spiral lamina away from the base, which was also the closest fiber to the stimulating electrode. The electric potential **(B)**, first **(D)** and second **(F)** derivatives on the right column were calculated on the fiber 10.15 mm away from the base.

The removal of fine structures in modiolar bone altered the electric potential along the cochlear ducts only slightly. Compared to ORI, a similar profile but with a downshift in value was observed in the electrical potential at the neural fiber tips in SIM, as shown in Figure 6A. The approximated basal decay rate for SIM was 0.17 dB/mm, which was marginally smaller than ORI. The comparison of the electrical potential along the entire length of ANFs revealed a more complex pattern: for many ANFs, as presented in Figures 8A,B as well as in Figures 9A,B, the potential drop along the fiber was smoother in SIM; as a result, the potential value on these fibers was initially larger in ORI, but as it traveled farther away from the spiral lamina, the potential value in SIM surpassed that in ORI. This “intersection” also varied slightly depending on the location of the stimulation electrode as well as that of the fiber. Nevertheless, the difference in ANF electric potentials between ORI and SIM was relatively small, where the maximal absolute difference only reaching up to 10%.

**Figure 10:**
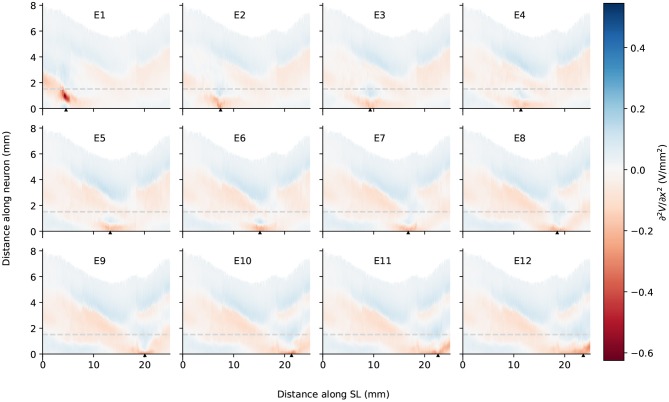
The second spatial derivative of electrical potential along the fiber direction within the simplified cochlear model. The stimulating electrode for each plot is specified at the top, and its location along the edge of spiral lamina is marked by the black solid triangles on the *x*-axis. The gray line in each plot indicates the soma location. “SL” stands for the outer edge of osseous spiral lamina.

In spite of small RDs in ANF electric potentials between ORI and SIM, the comparison of first and second derivatives of electric potential along the fiber, which are relevant for the initiation of action potentials, revealed a different story. Figures 8, 9 also presented the first and second derivatives of electric potentials along example fibers in both ORI and SIM, when the stimulating electrode was E3 and E5, respectively. Considerable fluctuations were found on the first and second derivatives with both models and their peaks were located at different locations along the fibers. For the exemplary fibers, maxima (and minima) in the first derivative between ORI and SIM differed by a factor of up to 1.5. In the case of the second derivative, such differences reached values up to 2; in addition, at several parts along the fibers, they had an opposite sign compared to those of SIM at the same location. The difference in the polarity/sign of the second spatial derivative can be clearly observed by comparing Figures 7, 10. As shown in Figure 7, major peaks occurred at regions in the proximity to stimulating electrodes. Specifically, negative peaks appeared at the synaptic ending of peripheral axons, soma, as well as peripheral and/or central axons close to the soma, whereas positive peaks showed up predominantly on the central axons. In comparison, major negative peaks in the SIM model were found, as revealed in Figure 10, only at the synaptic ending of peripheral axons, and the region around the soma (close to stimulating electrodes) exhibited mainly positive values for the second derivative. Nevertheless, derivatives of both models eventually converged toward the distal end of central axons, where the nerve trunk is solid in the ORI model; for example, as illustrated in Figure 8E, the convergence of second derivatives of the two models on the fiber closest to the stimulating electrode happened at approximately 4 mm away from the spiral lamina.

## 4. Discussion

In this study we presented a detailed-segmented FE cochlear model reconstructed from the μCT scans of a human cadaveric temporal bone; as the porous characteristics of the modiolar bone were carefully delineated during the segmentation, the microstructures of the auditory nerve were also included in the model. Moreover, we developed a new algorithm to reconstruct the auditory nerve fiber model. Due to the presence of irregular microstructures included in the FE model, a straight-forward spline interpolation as in previous modeling studies can inadvertently place segments of the nerve fibers outside the auditory nerve. By adopting a self-directed path-tracing through the edges of the FE auditory nerve mesh, we were able to ensure that the fiber tracts stayed within the auditory nerve, but not in any other structure of the model; and as the fibers inevitably passed through Rosenthal's canals in bundles, they displayed an appearance as natural as the osmium tetroxide-stained ANFs in the literature (Glueckert et al., 2018; van den Boogert et al., 2018).

To improve the lifelikeness of reconstructed ANFs, the following aspects should be taken into consideration. It has been reported in the literature on the anatomy of the auditory nerve in the cochlea that, due to the spiral feature of ANF bundles (Arnesen and Osen, 1978; Middlebrooks and Snyder, 2007), the peripheral axon of ANFs takes a radial trajectory from the organ of Corti to the corresponding region of spiral ganglion somas only within 20–60% relative length of the organ of Corti; fibers outside this region, i.e., in the most basal and apical regions, exhibit a more tangential course (Stakhovskaya et al., 2007; Li et al., 2019). The 45°-rotation of the projected spiral prior to ANF reconstruction was to generate a spiral “wrap” of ANF bundles; as a result, basal fibers managed to exhibit a more tangential trajectory. Furthermore, the ANF density is not uniform between base and apex and is highest in the middle region (Spoendlin and Schrott, 1989). Due to the non-uniform distribution, it is thus difficult to provide realistic representation without further information. Another missing key feature in the reconstructed fibers is the cell body, as cell bodies are considerably thicker than axons and therefore cannot be bundled up as tightly as the modeled nerve fibers. The fiber bundles should thus expand around to make room for the cell bodies. However, this is also difficult to achieve without knowing how the somas are packed in Rosenthal's canal. Therefore, further improvements to our algorithm are necessary to reconstruct more realistic ANFs, for instance, through the combination with imaging data of osmium tetroxide-stained fibers.

It has been established that a major problem for speech perception with CIs is the cross-talk between stimulating electrodes; this is because the electrical potential has a slow decay as it moves away from the stimulating electrode. This phenomenon thus leads to the lack of spatial selectivity in representing the frequency components of the sound source. In the present study, we found that the electrical potential decay in the auditory nerve was different depending on the location of the electrode in the cochlea and the direction of the decay. In general, the maximal potential value at the synaptic ending of peripheral axons became larger as the stimulating electrode reached into the apex, and the potential decay toward the base of the cochlea was faster than toward the apex; this agrees with the observations of electric potential in the scala tympani in Girzon (1987) and current density in Rosenthal's canals in Whiten (2007), despite the ground in these two modeling studies was placed much closer to the stimulating electrode. In addition, regardless of the electrode location, the decay toward the base shared more or less the same rate at 0.18 dB/mm; on the other hand, the decay toward the apex depended on the location of the electrode: the deeper it went into the cochlea, the earlier it flattened out. A similar trend was observed in the peak values of the second derivative as shown in Figure 7. Therefore, as the electrode shifted toward the apex, it recruited more fibers. This suggest that it may be beneficial to use a CI with uneven-spaced electrodes—an increasing distance between implant electrodes as moving toward the tip. It should nevertheless be noted that the analysis of the electrical potential alone does not predict the activation of nerve fibers. In the case of the activation of auditory nerve with a CI, where the cell bodies are relatively close to the stimulating electrodes, only cable models are able to predict threshold, polarity dependence and initiation site of the axon potential generation. Detailed analysis with the inclusion of a cable model to simulate ANFs will be performed in future research.

Apart from providing benefit to reconstruct lifelike ANFs, the fine modiolar details in the model may also have a major impact for predicting the activation pattern of ANFs. Our simulation results revealed that potentials along the cochlear duct and also along the nerve fibers were not much altered using the simplified model. However, spikes are initiated at the maxima of the activating function, i.e., the second derivative of the potential along an axon (if the axon is homogeneous) (Rattay, 1986), where we found substantial differences between the fine-segmented and simplified models. In the detailed model, the absolute values of the activating function were usually larger, which predicted lower thresholds, and more importantly, maxima occurred at different locations, which predicted different spike initiation sites. Even opposite signs were found for the values of second derivatives between ORI and SIM at several parts along the fibers, e.g., the initial segment of peripheral axon in the proximity of stimulating electrode, which suggested different polarity sensitivity; hence, while one model predicts depolarization and spike initiation at a given location, the other may instead predict a hyperpolarization. We therefore conclude that it is necessary to reconstruct a computational cochlear model with a detailed-segmented geometry combined with a detailed model of the neurons, which include dendrite, soma, and axon, to provide accurate predictions of ANF activation.

In order to confirm that no FE discretization error influenced the simulation outcomes, we generated two additional FE mesh using the detailed-segmented model, with 18,163,954 as well as 46,170,857 elements. The maximal absolute RD to the mesh with 21,937,778 elements for the coarser and finer models were 0.271 and 0.056%, respectively. This indicates the mesh used in this study was well-converged. Since it was difficult to retrieve information on the soft tissues from the μCT scans, the inner structure of the cochlea was not fully represented, and the blood vessels were included in the segmented nerve mesh model. A cochlear model including more tissue compartments, such as in Wong et al. (2015), is likely to provide more accurate prediction on the voltage profile. Nevertheless, our model was validated against intra-cochlear measurements from 16 implanted electrodes, and simulation results fell within the range of measurements, and in general presented a similar shape. This already indicates a good degree of validity for the model, especially considering that the cochlear structure was not fully represented, and the electrical properties were taken from literature without being fitted. At the same time, our measurement also presented several limitations: We were not capable of detecting individual disturbances to the electrode array, such as reduced contact due to scaring or tissue growth, and the presence of air bubbles; we were also unable to assess the size of patient cochleae or the exact placement of the electrode array. Future work on the model will involve incorporating more tissue compartments in order to investigate the sensitivity of electric potential and neural activation to the inclusion and variation of these properties.

## Data Availability Statement

The datasets generated for this study are available on request to the corresponding author.

## Ethics Statement

The studies involving human participants were reviewed and approved by Klinikum rechts der Isar (Munich, 2126/08). The patients/participants provided their written informed consent to participate in this study.

## Author Contributions

SB contributed to study design, FE model reconstruction, ANF reconstruction, model simulation, data analysis, and manuscript drafting. JEn contributed to study design, ANF reconstruction, data analysis, and manuscript drafting. MO-L and JEb contributed to intra-cochlear potential measurements and manuscript revising. RW and FS contributed to FE model reconstruction and manuscript revising. FB contributed to μCT image acquisition and manuscript revising. WH contributed to study design and critical manuscript revising. The final manuscript has been approved by all authors.

### Conflict of Interest

The authors declare that the research was conducted in the absence of any commercial or financial relationships that could be construed as a potential conflict of interest.
